# In Vitro Anticancer Activity and Mechanism of Action of an Aziridinyl Galactopyranoside

**DOI:** 10.3390/biomedicines10010041

**Published:** 2021-12-25

**Authors:** Estefanía Burgos-Morón, Nuria Pastor, Manuel Luis Orta, Julio José Jiménez-Alonso, Carlos Palo-Nieto, Margarita Vega-Holm, José Manuel Vega-Pérez, Fernando Iglesias-Guerra, Santiago Mateos, Miguel López-Lázaro, José Manuel Calderón-Montaño

**Affiliations:** 1 Department of Pharmacology, Faculty of Pharmacy, University of Seville, 41012 Seville, Spain; eburgos1@us.es (E.B.-M.); jjalonso@us.es (J.J.J.-A.); 2 Department of Cell Biology, Faculty of Biology, University of Seville, 41012 Seville, Spain; npastor@us.es (N.P.); morta2@us.es (M.L.O.); smateos@us.es (S.M.); 3 Department of Organic and Medicinal Chemistry, Faculty of Pharmacy, University of Seville, 41012 Seville, Spain; carlos.nieto@angstrom.uu.se (C.P.-N.); mvegaholm@us.es (M.V.-H.); vega@us.es (J.M.V.-P.); iglesias@us.es (F.I.-G.); 4 Department of Materials Science and Engineering, Nanotechnology and Functional Materials, Uppsala University, 751 03 Uppsala, Sweden

**Keywords:** aziridine, cancer, cytotoxic, cytotoxicity, selectivity, nucleotide excision repair

## Abstract

We recently screened a series of new aziridines β-D-galactopyranoside derivatives for selective anticancer activity and identified 2-methyl-2,3-[*N-*(4-methylbenzenesulfonyl)imino]propyl 2,3-di-O-benzyl-4,6-O-(S)-benzylidene-β-D-galactopyranoside (AzGalp) as the most promising compound. In this article, we explore the possible mechanisms involved in the cytotoxicity of this aziridine and evaluate its selective anticancer activity using cancer cells and normal cells from a variety of tissues. Our data show that AzGalp induces DNA damage (comet assay). Cells deficient in the nucleotide excision repair (NER) pathway were hypersensitive to the cytotoxicity of this compound. These results suggest that AzGalp induces bulky DNA adducts, and that cancer cells lacking a functional NER pathway may be particularly vulnerable to the anticancer effects of this aziridine. Several experiments revealed that neither the generation of oxidative stress nor the inhibition of glycolysis played a significant role in the cytotoxicity of AzGalp. Combinations of AzGalp with oxaliplatin or 5-fluorouracil slightly improved the ability of both anticancer drugs to selectively kill cancer cells. AzGalp also showed selective cytotoxicity against a panel of malignant cells versus normal cells; the highest selectivity was observed for two acute promyelocytic leukemia cell lines. Additional preclinical studies are necessary to evaluate the anticancer potential of AzGalp.

## 1. Introduction

Cancer kills millions of people every year, with metastasis being the main cause of cancer-related deaths [1]. When the disease spreads to other parts of the body, cancer cells are no longer localized and are difficult to eliminate by surgery or radiotherapy. At this stage of the disease, pharmacotherapy usually becomes the main form of treatment. However, available anticancer drugs usually do not cure the disease due to their limited selectivity toward cancer cells. The low efficacy of existing anticancer drugs is reflected in the poor survival rates of patients diagnosed with the most common metastatic cancers. For example, more than 50% of people diagnosed with lung cancer have distant metastasis at the time of diagnosis, and only 6% of them survive five years after diagnosis. Metastasis will continue to be an incurable disease for most patients until the development of therapies with high selectivity against cancer cells [1,2].

**Figure 1:**
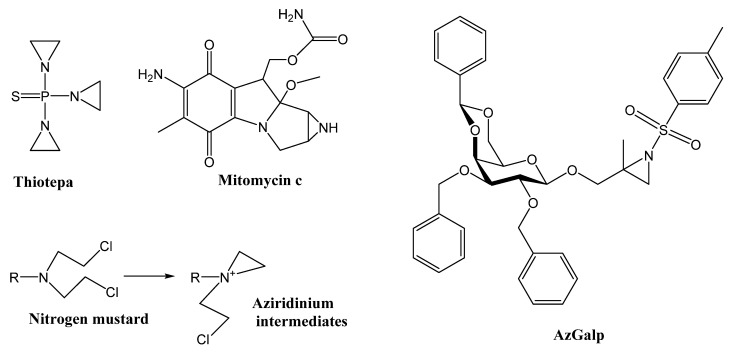
Chemical structure of several aziridine-containing compounds: Thiotepa, mitomycin c, aziridinium intermediates from activation of nitrogen mustard and 2-Methyl-2,3-[*N-*(4-methylbenzenesulfonyl)imino]propyl 2,3-di-O-benzyl-4,6-O-(S)-benzylidene-β-D-galactopyranoside (AzGalp).

Aziridines are highly reactive compounds based on a three-membered heterocycle similar to epoxide but with a nitrogen atom instead of an oxygen. The aziridine group is present in the structure of useful reaction intermediates in organic chemistry and also in a variety of secondary metabolites from plants and microorganisms [3,4]. Many of these compounds have shown anticancer activity [5,6,7,8]. In fact, some of them have been used in cancer therapy for decades, such as thiotepa and mitomycin C (Figure 1). Thiotepa (tris(1-aziridinyl)phosphine sulfide) is an alkylating agent approved for the treatment of breast, ovary and bladder cancer. While the main mechanism of this drug remains unclear, it is thought that the aziridyl groups of thiotepa induce crosslinks with DNA, interfering with DNA replication and cell division [9]. Mitomycin C is an aziridine alkaloid isolated from *Streptomyces* bacteria [3,10]. This anticancer drug is useful for the treatment of gastric and pancreatic adenocarcinomas. It is known that its aziridine moiety plays an important role in its cytotoxicity [10]. The cytotoxic mechanism of this alkaloid includes the alkylation of DNA, the generation of oxygen radicals and the inhibition of DNA and RNA synthesis. Other aziridine alkaloids isolated from nature have also shown anticancer activity [3]. These data suggest that compounds containing aziridine in their structure could have potential anticancer activity.

Previously, we carried out a preliminary screening of a new series of aziridines from alkenyl-β-D-galactorpyranoside derivatives for selective cytotoxic activity against the A549 human lung cancer cell line and the MRC-5 human lung normal cell line using the MTT assay [4]. Several aziridine-containing compounds showed notable selectivity against the A549 lung cancer cell line. The most selective compound, 2-methyl-2,3-[*N-*(4-methylbenzenesulfonyl)imino]propyl 2,3-di-O-benzyl-4,6-O-(S)-benzylidene-β-D-galactopyranoside (AzGalp, Figure 1), was approximately six times more cytotoxic against A549 lung cancer cells than against MRC-5 lung normal cells. In this article, we have evaluated the possible mechanisms involved in the cytotoxic activity of AzGalp. We have also assessed its cytotoxicity against a panel of normal and malignant cell lines.

## 2. Materials and Methods

### 2.1. Chemicals

Catalase, camptothecin (CPT), 4′,6-diamidino-2-phenylindole (DAPI), dichloroacetate (DCA), etoposide, 5-fluorouracil (5-FU), hydrogen peroxide (H_2_O_2_), 3-(4,5-dimethylthiazol-2-yl)-2,5-diphenyltetrazolium bromide (MTT), *N-*acetylcysteine (NAC), oxaliplatin and resazurin were purchased from Sigma (Merck KGaA, Darmstadt, Germany). Mn(III) tetrakis (1-methyl-4-pyridyl) porphyrin pentachloride (MnTMPyP) was obtained from Biomol International (Hamburg, Germany). The mounting medium for fluorescence (Vectashield) was purchased from Vector Laboratories (Burlingame, CA, United States); 2-Methyl-2,3-[*N-*(4-methylbenzenesulfonyl)imino]propyl 2,3-di-O-benzyl-4,6-O-(S)-benzylidene-β-D-galactopyranoside (AzGalp) was synthesized as previously described [4]. All other compounds used in this work were obtained from Sigma (Merck KGaA, Darmstadt, Germany). Stock solutions of AzGalp, CPT, etoposide, 5-FU, MnTMPyP and oxaliplatin were prepared in DMSO. Catalase, DCA, H_2_O_2_, MTT, NAC and resazurin were dissolved in culture medium before use.

### 2.2. Cell Lines

A549 (human non-small cell lung cancer cells) and MRC-5 (human lung fibroblastic cells) were purchased from the European Collection of Cell Cultures (Porton Down, Salisbury, UK). BJ-hTERT (hTERT immortalized foreskin fibroblast BJ cells), BJ-SV40T (SV40T-transformed BJ-hTERT cells) and BJ-RASV12 (HRASV12-transformed BJ-SV40T cells) were kindly provided by Dr. Hahn (Dana-Farber Cancer Institute, Boston, MA, USA) [11]. HeLa (human cervical carcinoma cells), HepG2 (human hepatocellular carcinoma cells), HL-60 (human acute promyelocytic leukemia cells), NB4 (human acute promyelocytic leukemia cells), PC3 (human prostate cancer cells), SW480 (human colon adenocarcinoma cells), U2OS (human osteosarcoma cells) and VH10 (human foreskin fibroblast cells) were generously provided by Dr. Helleday (Karolinska Institute, Stockholm, Sweden) [12]. UACC-62 (human melanoma cells) was obtained from the National Cancer Institute (NCI; Bethesda, MD, USA). To study the possible DNA damage response induced by AzGalp, the following parental and DNA repair-deficient cell lines were used: AA8 (parental Chinese hamster ovary cells, DNA repair proficient), UV4 (AA8 cells mutated in ERCC1, nucleotide excision repair (NER) deficient) and UV5 (AA8 cells mutated in ERCC2 (XPD), NER deficient). AA8, UV4 and UV5 were a gift from Dr. Helleday (Karolinska Institute, Stockholm, Sweden) [13,14]. A549, AA8, BJ-hTERT, BJ-RASV12, BJ-SV40T, HeLa, HepG2, MRC-5, SW480, U2OS, UACC-62, UV4, UV5 and VH10 were grown in Dulbecco’s modified Eagle medium (DMEM) high glucose medium. PC3 was cultured in DMEM-F12. HL60 and NB4 were kept as suspension cultures in RPMI 1640. All media were supplemented with 10% fetal bovine serum, 100 U/mL penicillin and 100 μg/mL streptomycin. All cells were maintained at 37 °C in a humidified atmosphere containing 5% CO_2_. Cell culture reagents were purchased from Thermo Fisher Scientific (Basel, Switzerland).

### 2.3. Cell Viability Assays

Exponentially growing cells were seeded in 96-well plates and allowed to attach and grow for 24 h. The cells were then treated with increasing concentrations of the tested drugs. At the end of the treatment period, cell viability was measured with the MTT assay or the resazurin assay, two widely used techniques.

In the case of the MTT assay, this technique detects viable cells using the ability of these cells to reduce the yellow tetrazolium MTT to an insoluble colored formazan product. Subsequently, this product was solubilized and analyzed by spectrophotometry. Dead cells cannot reduce the MTT into the colored formazan product. Briefly, 24 h after plating, cells were treated with the tested compounds for 48 h; except in experiments with DNA repair-proficient and -deficient cell lines, in which cells were only exposed for 24 h to drugs and allowed to grow for an additional 48 h in drug-free medium to allow them to repair possible DNA damage induced by the tested drugs. After the treatment period, the medium was removed, the cells were washed once with PBS and 125 μL MTT (1 mg/mL in medium) was added to each well. Plates were incubated for 2–4 h and then 80 μL 20% SDS in 0.02 M HCl was added to each well. Finally, plates were incubated overnight at 37 °C and optical densities were measured at 540 nm using an absorbance spectrophotometer microplate reader.

The resazurin assay is a redox-based colorimetric/fluorometric technique based on the ability of viable cells to reduce the blue compound resazurin into the pink, fluorescent and soluble product resorufin. The amount of resorufin produced is generally proportional to the number of living cells. In this assay, 24 h after seeding, cells were exposed to the tested drugs for 72 h. Then 100 μL resazurin in medium was added to each well (final concentration of 10 μg/mL) and, one hour later the fluorescence intensity was measured at 530/590 nm (excitation/emission) using a fluorescence microplate reader.

In both assays, cell viability was expressed as percentage compared to untreated cells. Results were expressed as the means ± standard error of the mean (SEM). All data are from at least three independent experiments.

### 2.4. Survival Assay

Cell survival after treatment was determined by clonogenic assay. AA8 and UV4 cells were seeded in 6 cm Petri dishes 4 h prior to treatment for 24 h. After that, drugs were removed and cells were allowed to form colonies for eight days. The colonies were then stained with 0.4% methylene blue in methanol for 15 min. Surviving colonies made up of more than 50 cells per colony were counted. Results were represented as percentage of survival referred to untreated cells.

### 2.5. Comet Assay

The single cell gel electrophoresis assay, also known as comet assay, is a widely used technique for the study of levels of damage in cellular DNA. In this assay, individual cells embedded in agarose are lysed, electrophoresed, stained with a fluorescent dye and examined under an epifluorescence microscope. A look through the microscope shows that DNA damaged cells have the appearance of a ‘comet’, with a head (undamaged DNA nucleoid part) and tail (DNA fragments). The quantity of DNA present in the tail is indicative of DNA damage. This assay has been described in detail by Singh et al. [15]. We followed this protocol with minor modifications previously described [16]. Briefly, standard slides were immersed in 1% normal melting agarose at 55 °C, left to allow the agarose to solidify and kept at 4 °C until use. Cells were seeded in 6-well plates, allowed to grow for 24 h and then cells were exposed to AzGalp or CTP (positive control) for 4 h. At the end of the treatment, cells were harvested by trypsinization, washed with PBS and resuspended in PBS at a concentration of approximately 1 million cells/mL; 10 µL cell suspension was mixed with 85 μL of low-melting agarose (LMA) at 37 °C and the mixture was quickly added to the slides with the first layer of agarose, spread using a coverslip and kept at 4 °C for 8 min to allow the LMA to solidify. The coverslips were then removed and another layer of 100 μL of LMA at 37 °C was added, covered with a coverslip and allowed to solidify at 4 °C for 8 min. After removing the coverslips, the slides were incubated in the dark for 1 h at 4 °C in a lysis buffer (pH 10.0) containing 0.25 M NaOH, 10% DMSO, 1% Triton X-100, 10 mM Tris-HCl, 100 mM Na2-EDTA and 2.5 M NaCl. Next, alkaline denaturation with electrophoretic buffer (300 mM NaOH and 1 mM Na2-EDTA) was carried out in an electrophoresis chamber for 20 min and then electrophoresis was performed at 1 V/cm for 20 min. The slides were later neutralized with 3 × 5 min washes of neutralizing buffer (0.4 M Tris-HCl, pH 7.5). Cells were stained with DAPI in Vectashield and images were taken with an epifluorescence microscope. The analysis of approximately 50 cells/sample was performed with the CometScore software. DNA damage levels were quantified for each cell and expressed as percent of DNA in the tail and as the tail moment (defined as the product of the tail length and the fraction of total DNA in the tail). The results were averaged from two independent experiments and expressed as mean ± SEM.

### 2.6. Glycolysis Inhibition

Inhibition of glycolysis was determined by measuring glucose consumed (initial product of glycolysis) and lactate produced (final product of glycolysis) in untreated and treated cells. Cells were seeded in 6-well plates at a density of 10^6^ cells/well and were allowed to attach for a few hours before treatment with the tested compounds for 8 h. After treatment, medium was recollected and glucose and lactate concentrations were determined using the Accutrend^®^ Plus analyzer together with Accutrend glucose strips and BM-Lactate strips (Roche Diagnostics, Barcelona, Spain). After calibrating the instrument with glucose and lactate calibration strips, test strips were used to determine glucose and lactate levels through colorimetric oxidase mediator reactions according to the manufacturer’s instructions [17]. The results are expressed as percentage of glucose consumption and percentage of lactate production in relation to untreated cells. Data were averaged from two independent experiments and shown as mean ± SEM.

### 2.7. Statistical Analysis

For statistical analysis, the *t*-test (paired, two-tailed) was used. A *p* value > 0.05 is not considered statistically significant and is not represented by any symbol. A *p* value < 0.05 is considered to correspond to statistical significance and is indicated with an asterisk (*), a *p* value < 0.01 is indicated with a double asterisk (**) and a *p* value < 0.001 is indicated with a triple asterisk (***). When the cytotoxic activity of a drug was determined against two cell lines, statistical analysis was carried out to compare the cytotoxicity of a particular concentration of the compound between both cell lines.

## 3. Results

### 3.1. AzGalp Induces DNA Damage

**Figure 2:**
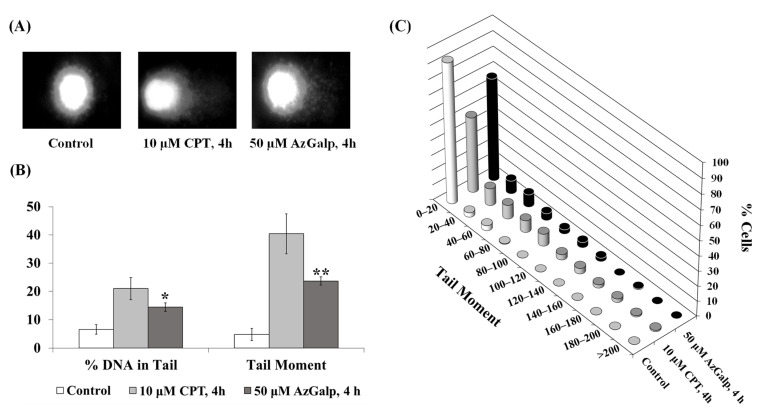
Determination of AzGalp-induced DNA damage in AA8 cells by the comet assay. (**A**) Representative photographs of control cells and cells exposed to 50 µM AzGalp or 10 µM camptothecin (CPT) for 4 h; (**B**) Quantification of DNA damage expressed as percentage of DNA damage in Tail and as Tail Moment (Tail length × percentage of DNA in the Tail); (**C**) Distribution of cells in the different intervals of values of Tail Moments. Data are averaged from two independent experiments. Statistical analysis was calculated using the paired *t*-test; * indicates *p* < 0.05, ** indicates *p* < 0.01.

Because aziridine-containing compounds are known to induce DNA damage [9,10], we used the comet assay to test whether AzGalp, the most interesting aziridine from our previous screening [4], could induce DNA damage. To obtain an appropriate concentration for the DNA damage assay, AA8 cells were exposed to various concentrations of AzGalp for 24 h and then allowed to grow in drug-free medium for 48 h. The percentage of cell viability was determined by the MTT assay; the IC50 value (mean ± SEM) was 397.0 ± 142.1 µM. We chose 50 µM AzGalp (a concentration below the IC50 value) and a 4-h exposure time for the comet assay. At this concentration and exposure time, the DNA damage observed in the comet assay is not caused by the DNA fragmentation occurring during cell death. AA8 cells were exposed to 50 µM AzGalp or 10 µM camptothecin (CPT, a known DNA-damaging agent used as a positive control) for 4 h, and the comet assay was performed to evaluate DNA damage. The results, represented in Figure 2, show that approximately 30% of cells treated with AzGalp developed higher levels of DNA damage than untreated cells. However, the level of DNA damage induced by AzGalp was lower than that of the DNA-damaging agent CPT. We tested another cytotoxic compound at the same time that did not show DNA damage in the comet assay (Figure S1).

### 3.2. Role of Nucleotide Excision Repair in the Cytotoxicity of AzGalp

**Figure 3:**
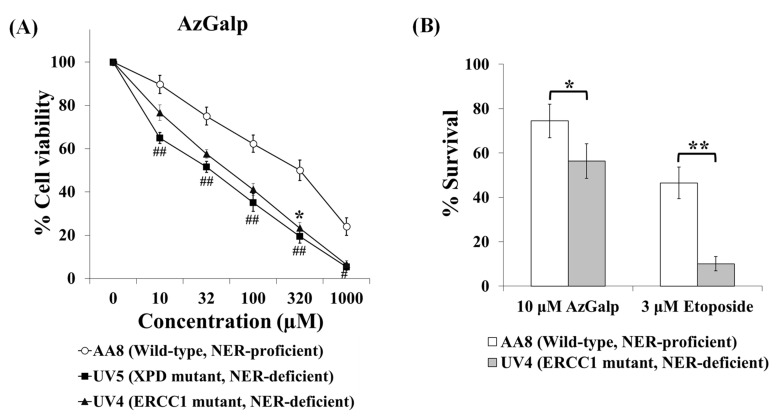
Cells deficient in nucleotide excision repair (NER) are hypersensitive to the cytotoxicity of AzGalp. (**A**) AA8 cells (NER proficient), UV5 (mutated in XPD; deficient in NER repair) and UV4 (mutated in ERCC1; deficient in NER repair) were treated with several concentrations of AzGalp for 24 h and, after a recovery period of 48 h, cell viability was determined with the MTT assay; (**B**) AA8 and UV4 cells were treated with AzGalp or etoposide (positive control) for 24 h. The cells were then allowed to form colonies in drug-free medium for seven days. Finally, the percentage of cell survival with respect to untreated cells was determined by the clonogenic assay. Student’s *t*-test was carried out to compare the cytotoxicity of a particular concentration of the compound between AA8 and UV4 (asterisks) or AA8 and UV5 (hashes). * indicates *p* < 0.05, ** indicates *p* < 0.01, # indicates *p* < 0.05, ## indicates *p* < 0.01.

Alkylating agents are known to induce bulky DNA adducts that distort the DNA double helix. This type of DNA damage is usually repaired by nucleotide excision repair (NER) [18,19]. Our next goal was to use NER-deficient cell lines to study whether this type of DNA damage was involved in the cytotoxicity of AzGalp. UV4 and UV5 cells (NER deficient) and AA8 cells (NER-proficient) were exposed to various concentrations of AzGalp for 24 h and, after 48 h in drug-free medium, cell proliferation was studied using the MTT assay. Figure 3A shows that both NER-deficient cell lines were hypersensitive to the cytotoxicity of this aziridine-containing compound. The IC50 values (means ± SEM; μM) were 318.7 ± 77.2; 58.6 ± 9.3 and 38.6 ± 6.3 for AA8, UV4 and UV5, respectively. These data show that NER-deficient cells were approximately six times more sensitive than NER-proficient cells. That hypersensitivity of NER-deficient cells was also observed when we performed the clonogenic assay (Figure 3B). UV4 and AA8 cells were treated with 10 µM AzGalp or 3 µM etoposide (positive control) for 24 h and then the cells were allowed to grow in colonies in drug-free medium for one week. UV4 cells were more sensitive to the cytotoxic effect of AzGalp or etoposide than AA8 cells. All these results suggest that AzGalp may induce DNA adducts, which can be repaired by the NER pathway.

### 3.3. Evaluation of Other Possible Mechanisms Involved in the Selective Cytotoxic Activity of AzGalp

**Figure 4:**
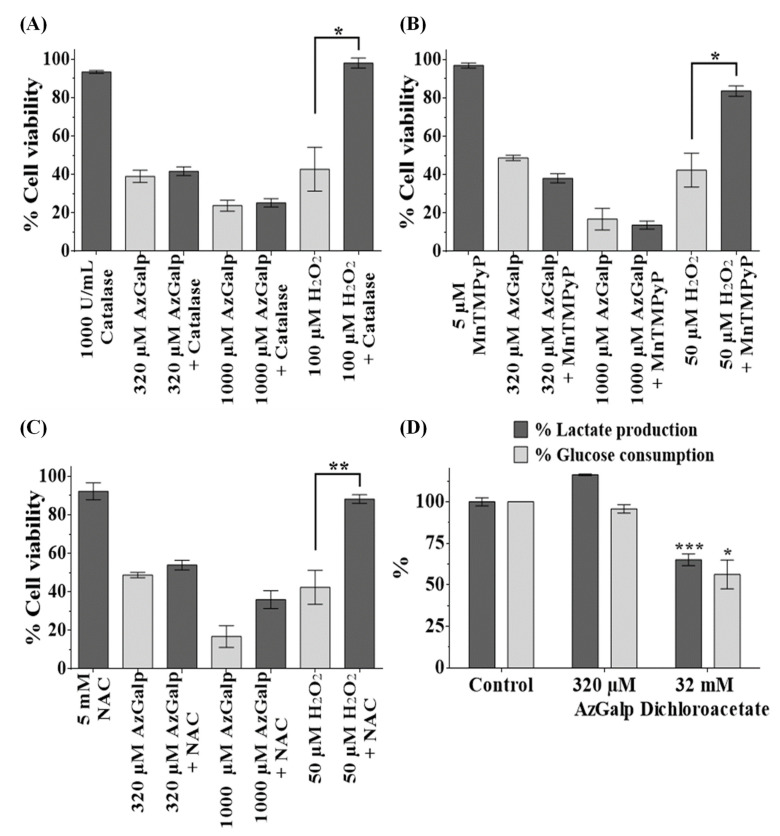
Neither the generation of reactive oxygen species nor the inhibition of glycolysis participate in the cytotoxicity of AzGalp. A549 cells were treated with AzGalp or H_2_O_2_ for 48 h in the absence or presence of catalase (**A**), superoxide dismutase mimetic MnTMPyP (**B**) and NAC (**C**). Antioxidants were added 1 h before AzGalp or H_2_O_2_. Cell viability was determined by the MTT assay; (**D**) Percentage of glucose consumed and percentage of lactate produced by A549 cells exposed for 8 h to AzGalp or Dichloroacetate compared to untreated cells. Statistical analysis was calculated using the paired *t*-test; * indicates *p* < 0.05, ** indicates *p* < 0.01, *** indicates *p* < 0.001.

Evidence suggests that reactive oxygen species (ROS) play an important role in the anticancer activity of several clinically useful drugs [20,21]. To test whether ROS generation participates in AzGalp-induced cytotoxicity, A549 cells were exposed to this compound in the presence or absence of the antioxidants catalase, *N-*acetylcysteine (NAC) and the superoxide dismutase mimetic MnTMPyP for 48 h and cell proliferation was determined with the MTT assay (Figure 4A–C). The cytotoxicity of H_2_O_2_ (positive control) decreased in the presence of the three antioxidants. Neither MnTMPyP nor catalase prevented the cytotoxicity of AzGalp. While NAC slightly prevented cytotoxic activity, it was not statistically significant. These data suggest that ROS formation does not participate in the anticancer activity of AzGalp.

Because cancer cells generally depend highly on glycolysis for proliferation and survival, inhibition of glycolysis can trigger selective anticancer effects [22,23]. To evaluate whether AzGalp could act as a glycolysis inhibitor, A549 were treated with AzGalp for 8 h and concentrations of glucose (initial product of glycolysis) and lactate (final product of glycolysis) were measured. The glycolysis inhibitor DCA was used as a positive control. DCA reduced lactate production and glucose consumption. However, AzGalp did not affect the glycolytic activity of A549 cells (Figure 4D), therefore indicating that the cytotoxic activity of this aziridine is not mediated by inhibition of the glycolytic pathway.

### 3.4. Cytotoxicity of AzGalp in Combination with the Anticancer Drugs 5-Fluorouracil and Oxaliplatin

**Figure 5:**
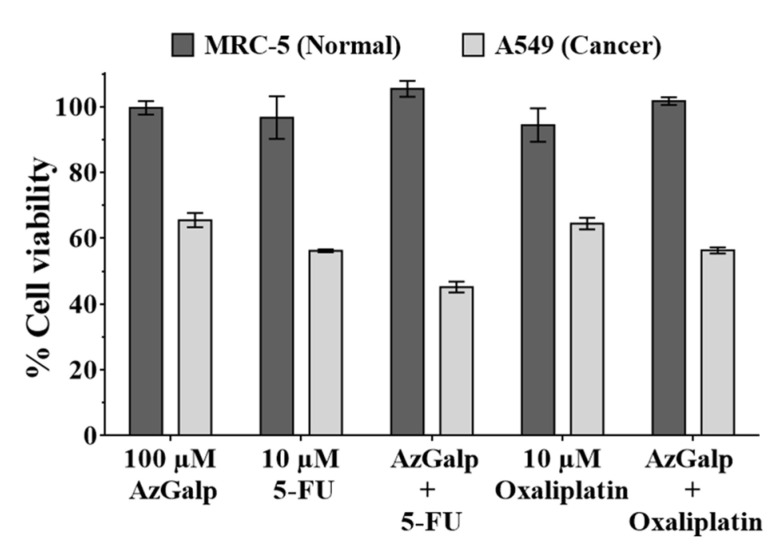
Cytotoxicity of AzGalp in combination with the anticancer drugs 5-fluorouracil (5-FU) and oxaliplatin. A549 lung cancer cells and MRC-5 lung normal cells were treated for 48 h with AzGalp alone or in combination with 5-FU or oxaliplatin. Cell viability was assessed by the MTT assay.

Chemotherapy regimens are usually based on the combination of anticancer drugs to improve their specificity and efficacy. Therefore, we tested the cytotoxic activity of AzGalp in combination with the anticancer drugs 5-FU (antimetabolite) and oxaliplatin (alkylating agent), two commonly used drugs to treat different types of cancer. MRC-5 and A549 cells were treated with 100 μM AzGalp with and without 10 μM 5-FU or 10 μM oxaliplatin for 48 h. All drugs were added simultaneously when tested in combination. Cell proliferation was determined by the MTT assay. Results are represented in Figure 5. While the combination of AzGalp with 5-FU or oxaliplatin killed more cancer cells than when these drugs were administered individually, we did not observe a clear synergistic effect. However, the combinations did not increase cytotoxicity against normal cells. Combinations of AzGalp with oxaliplatin or 5-fluorouracil slightly improved the ability of both anticancer drugs to selectively kill cancer cells.

### 3.5. Determination of the Cytotoxicity of AzGalp against a Panel of Cancer Cells

**Figure 6:**
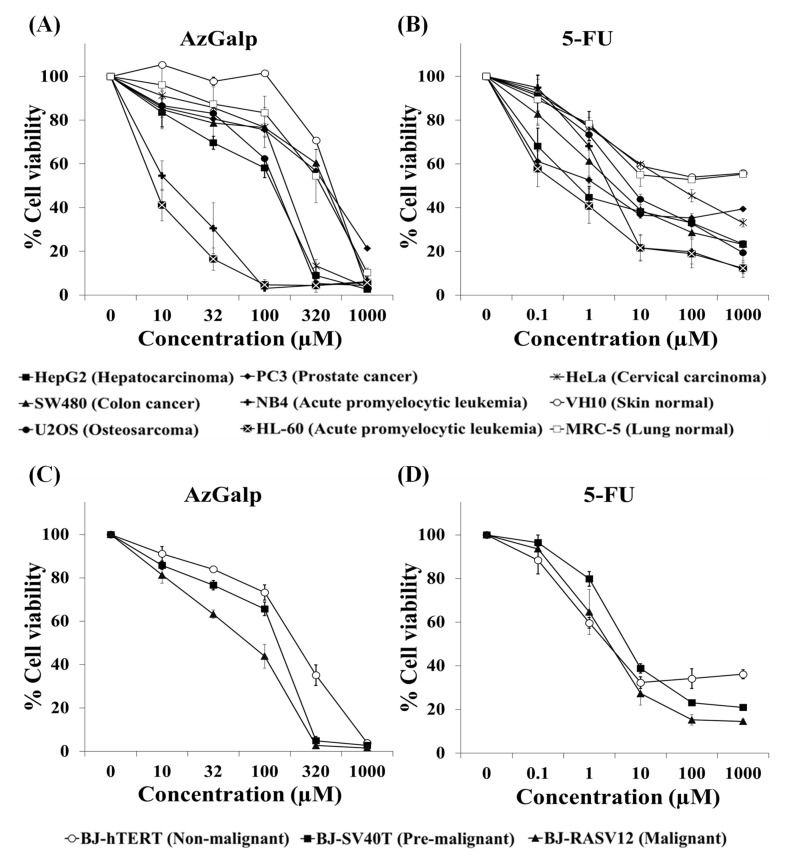
Evaluation of cytotoxic activity of AzGalp against five solid tumor cancer cell lines (HeLa, HepG2, PC3, SW480 and U2OS), two acute promyelocytic leukemia cell lines (HL-60 and NB4), two normal cell lines (MRC-5 and VH10) (**A**) and three genetically modified cell lines (BJ-hTERT, BJ-SV40T and BJ-RASV12) (**C**); 5-FU was used as a positive control (**B**,**D**). Cells were exposed to several concentrations of AzGalp or 5-FU for 72 h and cell viability was determined with the resazurin assay.

Next, we evaluated the cytotoxic activity of AzGalp in cancer cell lines derived from solid tumors (HeLa, PC3, U2OS, HepG2 and SW480) and from hematological malignancies (NB4 and HL-60). To study the selectivity against cancer cells, MRC-5 and VH10 cells were used as normal cell lines; 5-FU was used as a positive control. All cells were treated with AzGalp or 5-FU for 72 h and cell viability was evaluated with the resazurin assay. This assay was used to determine cell viability instead of the MTT assay because the HL60 and NB4 cell lines grow in suspension. Unlike the MTT assay, the resazurin assay does not require removal of culture medium. The results are collected in Figure 6A,B and Table 1. Leukemia cell lines were approximately 15 times more sensitive than normal cells to the cytotoxicity of our aziridine. AzGalp also showed selective cytotoxicity against liver cancer HepG2 cells and osteosarcoma U2OS cells, being approximately 2.5 times more cytotoxic against these cell lines than against normal cell lines. This compound showed a similar cytotoxic effect against HeLa cervical carcinoma cells, PC3 prostate cancer cells and SW480 colon adenocarcinoma cells than against MRC-5 and VH10 normal cells.

We also evaluated the selective cytotoxic activity of AzGalp against cancer cells using an in vitro model of malignant transformation [11,12]. This model consists of three genetically modified cell lines: BJ-hTERT, BJ-SV40T and BJ-RASV12. All cells are derived from normal foreskin BJ cells transformed genetically using hTERT (human telomerase reverse transcriptase), SV40LT (Simian virus 40 large T antigen) and HRAS. All of these cells express hTERT to avoid senescence. BJ-SV40T and BJ-RASV12 cells express SV40LT which inactivates two major tumor suppressors, RB and p53. BJ-RASV12 cells also express HRAS, an oncogene associated with numerous carcinogenic events. In this model, BJ-hTERT cells are considered as non-malignant, BJ-SV40T as pre-malignant and BJ-RASV12 as malignant cells. We observed that AzGalp was more cytotoxic against pre-malignant and malignant cells (Figure 6C). BJ-RASV12 malignant cells were three times more sensitive to AzGalp than BJ-hTERT non-malignant cells. It is worth mentioning that we did not observe this selectivity with the anticancer drug 5-FU (Figure 6D).

## 4. Discussion

Patients with metastatic cancers need selective anticancer treatments to improve their poor survival rates [1]. We previously screened a series of new aziridines β-D-galactopyranoside derivatives for selective anticancer activity and identified AzGalp as the most promising compound [4]. In this article, we study possible mechanisms involved in the cytotoxicity of this aziridine and evaluate its selective anticancer activity in additional cancer cells and normal cells.

It is known that aziridine-containing compounds (e.g., thiotepa and mitomycin C) and aziridinium intermediates formed during the activation of nitrogen mustards (structures in Figure 1) induce DNA alkylation damage [10]. The alkylation is due to the electrophilic character of the aziridine ring, which reacts with endogenous nucleophiles such as nitrogenous bases in DNA. This reaction generates DNA adducts that can block DNA replication and transcription machinery, generating DNA lesions and death cell [10]. The reactivity of the aziridine ring with DNA bases suggested that the cytotoxic effect of AzGalp could be due to the generation of DNA damage. Therefore, we began our investigation by studying the ability of AzGalp to induce DNA damage. To test this hypothesis, we used the comet assay, a classic DNA damage detection technique. We observed that cells treated with AzGalp for a short time showed high levels of DNA damage (Figure 2).

Our next objective was to evaluate whether AzGalp-induced DNA damage played a role in its cytotoxicity. Cells have developed several pathways to repair DNA lesions induced by either endogenous processes (e.g., replication) or exogenous sources (e.g., DNA-damaging drugs). It is known that the type of DNA lesion induced by alkylating agents is generated by DNA adducts, which distort the DNA double helix. These adducts are usually repaired by NER. Therefore, NER-deficient cells are generally more sensitive to alkylating agents, including aziridine-containing drugs [18,19]. Therefore, we evaluated the role of NER in the cytotoxic effect of AzGalp. The data in Figure 3 show that NER-deficient cells were hypersensitive to AzGalp, suggesting that this aziridine may induce DNA double helix distortion, which can be repaired by NER. The hypersensitivity of NER-deficient cells to AzGalp may have therapeutic implications. Evidences suggest that tumor cells have defects in DNA repair pathways that make them incapable of correctly repairing some types of DNA lesions [18,24]. These defects may explain why cancer cells are more sensitive than healthy cells to specific DNA-damaging drugs. For example, several studies suggest that the efficacy of platinum compounds and alkylating agents against specific tumors, such as testicular cancer and non-small-cell lung cancer, may be associated with defects in NER of these tumors [18]. Unlike healthy cells, these tumor cells would not be able to repair the type of DNA lesions induced by these drugs and would die. These data suggest that NER-deficient cancer cells may be more vulnerable to the cytotoxicity of AzGalp.

While the generation of DNA damage seems to play an important role in the cytotoxic activity of AzGalp, it may not be the only mechanism of action involved. It is known that anticancer drugs usually kill cancer cells through different pathways. For example, mitomycin C alkylates DNA, generates ROS and inhibits DNA, RNA and protein synthesis. We performed several assays to determine the possible participation of other mechanisms of action in the selective cytotoxic activity of AzGalp. The generation of ROS is a common cytotoxic mechanism of anticancer drugs [20,23,25,26,27,28]. ROS are involved in oxidative DNA damage, mitochondrial damage and apoptosis. To test the contribution of oxidative stress in AzGalp cytotoxicity, we evaluated its cytotoxic effect in the presence or absence of antioxidants (Figure 4). We did not observe a significant reduction in cytotoxicity in the presence of antioxidants, indicating that ROS do not participate in the anticancer activity of this aziridine compound. While NAC slightly prevented AzGalp cytotoxicity, this effect may not necessarily be mediated by a ROS-scavenging mechanism. NAC acts directly as an oxidant scavenger or indirectly as a precursor to gluthatione [29]. NAC could also protect against toxic agents by direct adduct formation. NAC has nucleophilic character and may react directly with electrophilic agents [30]. Therefore, NAC may chemically react with AzGalp, decreasing its cytotoxicity.

Next, we explored whether inhibition of glycolysis played a role in the selective cytotoxicity of AzGalp. During carcinogenesis, cancer cells undergo metabolic reprogramming, including a high dependence on glycolysis. This metabolic pathway is necessary to obtain energy and metabolic intermediates for macromolecular biosynthesis to support the high proliferation rate of cancer cells [31,32,33]. It is believed that inhibition of glycolysis could selectively kill cancer cells because they would not support metabolic reprogramming [22,23]. Because sugar analogues, such as 2-deoxy-D-glucose and 2-deoxy-D-galactose, are potent glycolytic inhibitors [22,34] and AzGalp is a β-D-galacto-pyranoside derivative, we tested whether this compound could inhibit glycolysis as a possible mechanism of its selective cytotoxicity. However, the treatment with AzGalp did not decrease either the consumption of glucose (the initial product of glycolysis) nor the production of lactate (the final product of glycolysis); these data indicate that the cytotoxicity of our aziridine is not mediated by an inhibition of the glycolytic pathway.

Since most anticancer drugs are generally used in combination, we evaluated the effects of the combination of AzGalp with other anticancer agents. Because alkylating agents and antimetabolites are widely used in cancer therapy, we evaluated the cytotoxic effect of the combination of AzGalp with the alkylating agent oxaliplatin and the antimetabolite 5-FU. We observed that the combination of AzGalp with these chemotherapeutic drugs killed more cancer cells and fewer normal cells than any of these drugs individually. However, this selectivity improvement was mild.

We previously reported that AzGalp showed selective cytotoxicity against lung cancer, breast cancer and melanoma cells [4]. In this study, AzGalp also showed selective cytotoxic activity against other solid cancer cell lines, such as liver cancer HepG2 cells and osteosarcoma U2OS cells, and against hematological malignancies, such as acute promyelocytic leukemia HL60 and NB4 cells. We compared the cytotoxic activity of AzGalp with the anticancer drug 5-FU. AzGalp displayed a better selective cytotoxic profile against acute promyelocytic leukemia cell lines than 5-FU. At concentrations between 10 and 100 µM, the viability of leukemia cells exposed to AzGalp was progressively reduced to 5%, while the viability of cells exposed to 5-FU remained higher than 20%. In the same concentration range, AzGalp did not alter the viability of normal cells, while 5-FU showed cytotoxicity against these cells.

Finally, we used three genetically modified skin cell lines with an increasing degree of malignancy to evaluate the selectivity of AzGalp. These cell lines are derived from normal BJ cells, which are altered in specific genes/proteins to progressively acquire the properties of cancer cells [11]. AzGalp was selectively cytotoxic to BJ-SV40T and BJ-RASV12 cells (Figure 6), suggesting that this aziridine compound has selective activity against malignant transformed cells. Malignant BJ-RASV12 cells express the oncogene HRAS and have inactive p53 and RB, two major tumor suppressors. Oncogene activation and tumor suppressor inactivation are frequently found in tumor cells and have been associated with high levels of basal DNA damage of cancer [18,35,36]. In addition, cancer cells usually have defects in DNA repair pathways that also contribute to their genomic instability. The high genomic instability and DNA repair defects in tumor cells make them more vulnerable than healthy cells to DNA-damaging agents. Unlike cancer cells, healthy cells have an operative DNA damage response that would allow them to repair DNA damage and survive exposure to these DNA-damaging drugs. This could explain why several DNA-damaging drugs currently used in clinic have moderate selective anticancer activity and prolong the survival of cancer patients. These data support the idea that the selective cytotoxicity of AzGalp against malignant cells could be mediated by its ability to induce DNA damage.

## 5. Conclusions

This article shows that the aziridine compound AzGalp has selective anticancer activity in vitro that is mediated, at least in part, by induction of DNA damage. It also shows that NER-deficient cells are significantly hypersensitive to the cytotoxicity of AzGalp; this suggests that cancer cells with deficiency in this DNA repair pathway could be hypersensitive to this compound. Our results suggest that AzGalp could have anticancer therapeutic potential because it showed higher cytotoxicity against several types of cancer cells than against non-malignant cells. Animal studies are necessary to test the efficacy and safety of AzGalp. This aziridine β-D-galactopyranoside derivative could also be a promising lead compound for the development of new anticancer drugs.

## Figures and Tables

**Table 1:** IC50 values of AzGalp and 5-FU against human non-malignant cell lines and human cancer cell lines. Cells were exposed to the drugs for 72 h and then cell viability was determined with the resazurin assay.

Cell Line	IC50 (Mean ± SEM, µM)	
	AzGalp	5-FU
**Normal Cells**		
MRC-5 (Human lung non-malignant cells)	341.1 ± 85.0	˃1000
VH10 (Human skin non-malignant cells)	410.9 ± 39.4	˃1000
**Cancer Cells**		
HeLa (Human cervical carcinoma cells)	191.0 ± 35.8	66.3 ± 27.0
HepG2 (Human hepatocellular carcinoma cells)	121.7 ± 12.4	0.6 ± 0.3
HL-60 (Human acute promyelocytic leukemia cells)	11.1 ± 0.9	0.9 ± 0.8
NB4 (Human acute promyelocytic leukemia cells)	21.4 ± 9.9	2.5 ± 0.3
PC3 (Human prostate cancer cells)	400.7 ± 14.8	1.5 ± 0.3
SW480 (Human colon adenocarcinoma cells)	387.8 ± 30.9	2.9 ± 1.3
U2OS (Human osteosarcoma cells)	120.5 ± 8.0	6.4 ± 0.7
**Genetically Modified Cells**		
BJ-hTERT (hTERT-immortalized skin non-malignant BJ cells)	208.7 ± 24.2	2.4 ± 0.5
BJ-SV40T (SV40T-transformed BJ-hTERT cells)	142.7 ± 8.1	5.4 ± 0.7
BJ-RASV12 (HRASV12-transformed BJ-SV40T cells)	76.3 ± 17.8	3.0 ± 1.4

## Data Availability

Not applicable.

## Supplementary Materials

The following are available online at https://www.mdpi.com/article/10.3390/biomedicines10010041/s1. Figure S1: Syringic acid does not induce DNA damage. AA8 cells were treated with the positive control camptothecin (CPT) or syringic acid for 4 h. After treatment, the comet assay was performed to detect DNA damage.

## References

[1] Siegel R.L., Miller K.D., Fuchs H.E., Jemal A. (2021). Cancer statistics, 2021. CA Cancer J. Clin..

[2] López-Lázaro M. (2015). A simple and reliable approach for assessing anticancer activity in vitro. Curr. Med. Chem..

[3] Ismail F.M.D., Levitsky D.O., Dembitsky V.M. (2009). Aziridine alkaloids as potential therapeutic agents. Eur. J. Med. Chem..

[4] Vega-Pérez J.M., Palo-Nieto C., Vega-Holm M., Gongora-Vargas P., Calderón-Montaño J.M., Burgos-Morón E., López-Lázaro M., Iglesias-Guerra F. (2013). Aziridines from alkenyl-beta-D-galactopyranoside derivatives: Stereoselective synthesis and *in vitro* selective anticancer activity. Eur. J. Med. Chem..

[5] Grüschow S., Chang L.C., Mao Y., Sherman D.H. (2007). Hydroxyquinone O-Methylation in Mitomycin Biosynthesis. J. Am. Chem. Soc..

[6] Hirayama N., Shirahata K. (1991). Structural studies of mitomycins. IV. Structure of albomitomycin A. Acta Crystallogr. Sect. C Cryst. Struct. Commun..

[7] Fiallo M.M.L., Deydier E., Bracci M., Garnier-Suillerot A., Halvorsen K. (2003). Mitomycin Antitumor Compounds. 2. Interaction of Transition Metal Ions with Mitomycin, C. Solution Structure and Biological Activity of a Pd^2+^–MMC Complex. J. Med. Chem..

[8] Coleman R.S., Kong J.S., Richardson T.E. (1999). Synthesis of Naturally Occurring Antitumor Agents:  Stereocontrolled Synthesis of the Azabicyclic Ring System of the Azinomycins. J. Am. Chem. Soc..

[9] Torabifard H., Fattahi A. (2013). DFT study on Thiotepa and Tepa interactions with their DNA receptor. Struct. Chem..

[10] Puyo S., Montaudon D., Pourquier P. (2014). From old alkylating agents to new minor groove binders. Crit. Rev. Oncol. Hematol..

[11] Hahn W.C., Counter C.M., Lundberg A.S., Beijersbergen R.L., Brooks M.W., Weinberg R.A. (1999). Creation of human tumour cells with defined genetic elements. Nature.

[12] Gad H., Koolmeister T., Jemth A.-S., Eshtad S., Jacques S.A., Ström C.E., Svensson L.M., Schultz N., Lundbäck T., Einarsdottir B.O. (2014). MTH1 inhibition eradicates cancer by preventing sanitation of the dNTP pool. Nature.

[13] Bryant H.E., Ying S., Helleday T. (2006). Homologous recombination is involved in repair of chromium-induced DNA damage in mammalian cells. Mutat. Res..

[14] Meschini R., Marotta E., Berni A., Filippi S., Fiore M., Mancinelli P., Natarajan A.T., Palitti F. (2008). DNA repair deficiency and BPDE-induced chromosomal alterations in CHO cells. Mutat. Res..

[15] Singh N.P., McCoy M.T., Tice R.R., Schneider E.L. (1988). A simple technique for quantitation of low levels of DNA damage in individual cells. Exp. Cell Res..

[16] Pastor N., López-Lázaro M., Tella J.L., Baos R., Hiraldo F., Cortes F. (2001). Assessment of genotoxic damage by the comet assay in white storks (*Ciconia ciconia*) after the Donana Ecological Disaster. Mutagenesis.

[17] Cao X., Bloomston M., Zhang T., Frankel W.L., Jia G., Wang B., Hall N.C., Koch R.M., Cheng H., Knopp M.V. (2008). Synergistic antipancreatic tumor effect by simultaneously targeting hypoxic cancer cells with HSP90 inhibitor and glycolysis inhibitor. Clin. Cancer Res..

[18] Calderón-Montaño J.M., Burgos-Morón E., Orta M.L., López-Lázaro M. (2014). Effect of DNA repair deficiencies on the cytotoxicity of drugs used in cancer therapy—A review. Curr. Med. Chem..

[19] Zheng H., Wang X., Warren A.J., Legerski R.J., Nairn R.S., Hamilton J.W., Li L. (2003). Nucleotide excision repair- and polymerase eta-mediated error-prone removal of mitomycin C interstrand cross-links. Mol. Cell. Biol..

[20] Martín-Cordero C., León-González A.J., Calderón-Montaño J.M., Burgos-Morón E., López-Lázaro M. (2012). Pro-oxidant natural products as anticancer agents. Curr. Drug Targets.

[21] Galadari S., Rahman A., Pallichankandy S., Thayyullathil F. (2017). Reactive oxygen species and cancer paradox: To promote or to suppress?. Free Radic. Biol. Med..

[22] Chen X., Li L., Guan Y., Yang J., Cheng Y. (2016). Anticancer strategies based on the metabolic profile of tumor cells: Therapeutic targeting of the Warburg effect. Acta Pharmacol. Sin..

[23] López-Lázaro M. (2010). A new view of carcinogenesis and an alternative approach to cancer therapy. Mol. Med..

[24] Weeden C.E., Asselin-Labat M.-L. (2018). Mechanisms of DNA damage repair in adult stem cells and implications for cancer formation. Biochim. Biophys. Acta–Mol. Basis Dis..

[25] Yokoyama C., Sueyoshi Y., Ema M., Mori Y., Takaishi K., Hisatomi H. (2017). Induction of oxidative stress by anticancer drugs in the presence and absence of cells. Oncol. Lett..

[26] Fang J., Nakamura H., Iyer A.K. (2007). Tumor-targeted induction of oxystress for cancer therapy. J. Drug Target..

[27] Simizu S., Takada M., Umezawa K., Imoto M. (1998). Requirement of caspase-3(-like) protease-mediated hydrogen peroxide production for apoptosis induced by various anticancer drugs. J. Biol. Chem..

[28] Trachootham D., Alexandre J., Huang P. (2009). Targeting cancer cells by ROS-mediated mechanisms: A radical therapeutic approach?. Nat. Rev. Drug Discov..

[29] Zafarullah M., Li W.Q., Sylvester J., Ahmad M. (2003). Molecular mechanisms of N-acetylcysteine actions. Cell. Mol. Life Sci..

[30] Nocca G., D’Antò V., Desiderio C., Rossetti D.V., Valletta R., Baquala A.M., Schweikl H., Lupi A., Rengo S., Spagnuolo G. (2010). N-acetyl cysteine directed detoxification of 2-hydroxyethyl methacrylate by adduct formation. Biomaterials.

[31] Altenberg B., Greulich K.O. (2004). Genes of glycolysis are ubiquitously overexpressed in 24 cancer classes. Genomics.

[32] Kunkel M., Reichert T.E., Benz P., Lehr H.-A., Jeong J.-H., Wieand S., Bartenstein P., Wagner W., Whiteside T.L. (2003). Overexpression of Glut-1 and increased glucose metabolism in tumors are associated with a poor prognosis in patients with oral squamous cell carcinoma. Cancer.

[33] Medina R.A., Owen G.I. (2002). Glucose transporters: Expression, regulation and cancer. Biol. Res..

[34] Laszlo J., Landau B., Wight K., Burk D. (1958). The effect of glucose analogues on the metabolism of human leukemic cells. J. Natl. Cancer Inst..

[35] Abulaiti A., Fikaris A.J., Tsygankova O.M., Meinkoth J.L. (2006). Ras Induces Chromosome Instability and Abrogation of the DNA Damage Response. Cancer Res..

[36] Gavande N.S., VanderVere-Carozza P.S., Hinshaw H.D., Jalal S.I., Sears C.R., Pawelczak K.S., Turchi J.J. (2016). DNA repair targeted therapy: The past or future of cancer treatment?. Pharmacol. Ther..

