# Supplementary material for: In Vitro Anticancer Activity and Mechanism of Action of an Aziridinyl Galactopyranoside
Source: Biomedicines. 2021 Dec 25;10(1):41. doi: 10.3390/biomedicines10010041 (PMC8773213; doi:10.3390/biomedicines10010041)
Supplement: Supplementary file 1 [file biomedicines-10-00041-s001.zip › biomedicines-1492808-supplementary.pdf]

biomedicines

MDPI

Article

In Vitro Anticancer Activity and Mechanism of Action of an Aziridinyl Galactopyranoside

Estefanía Burgos-Morón [1], Nuria Pastor [2], Manuel Luis Orta [2], Julio José Jiménez-Alonso [1], Carlos Palo-Nieto [3,4], Margarita Vega-Holm [3], José Manuel Vega-Pérez [3], Fernando Iglesias-Guerra [3], Santiago Mateos [2], Miguel López-Lázaro [1]*, José Manuel Calderón-Montaño [1]*

[1] Department of Pharmacology, Faculty of Pharmacy, University of Seville, 41012 Seville, Spain; eburgos1@us.es (E.B.-M.); jjalonso@us.es (J.J.J.-A.)
[2] Department of Cell Biology, Faculty of Biology, University of Seville, 41012 Seville, Spain; npastor@us.es (N.P.); morta2@us.es (M.L.O.); smateos@us.es (S.M.)
[3] Department of Organic and Medicinal Chemistry, Faculty of Pharmacy, University of Seville, 41012 Seville, Spain; carlos.nieto@angstrom.uu.se (C.P.-N.); mvegaholm@us.es (M.V.-H.); vega@us.es (J.M.V.-P.); iglesias@us.es (F.I.-G.)
[4] Department of Materials Science and Engineering, Nanotechnology and Functional Materials, Uppsala University, 751 03 Uppsala, Sweden
* Correspondence: mlopezlazaro@us.es (M.L.-L.); jcalderon@us.es (J.M.C.-M.)

Citation: Burgos-Morón, E.; Pastor, N.; Orta, M.L.; Jiménez-Alonso, J.J.; Palo-Nieto, C.; Vega-Holm, M.; Vega-Pérez, J.M.; Iglesias-Guerra, F.; Mateos, S.; López-Lázaro, M.; et al. In Vitro Anticancer Activity and Mechanism of Action of an Aziridinyl Galactopyranoside. *Biomedicines* **2022**, *10*, 41. https://doi.org/10.3390/biomedicines10010041

Academic Editors: Silvia Ortega-Gutierrez and María L. López-Rodríguez

Received: 18 November 2021
Accepted: 22 December 2021
Published: 25 December 2021

Publisher's Note: MDPI stays neutral with regard to jurisdictional claims in published maps and institutional affiliations.

Copyright: © 2021 by the authors. Licensee MDPI, Basel, Switzerland. This article is an open access article distributed under the terms and conditions of the Creative Commons Attribution (CC BY) license (https://creativecommons.org/licenses/by/4.0/).

1. SUPPLEMENTARY FIGURE

(A)

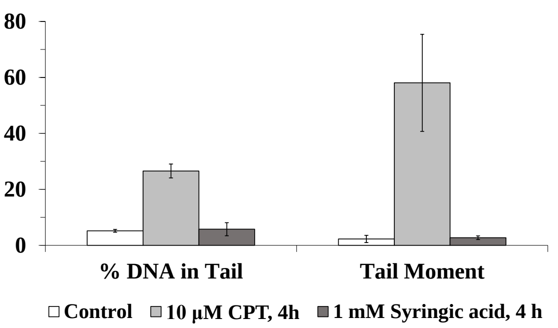

(B)

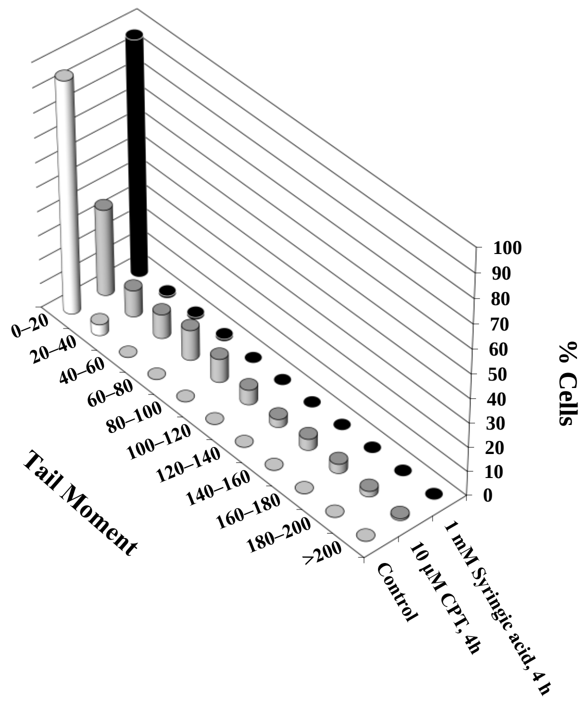

Figure S1. Syringic acid does not induce DNA damage. AA8 cells were treated with the positive control camptothecin (CPT) or syringic acid for 4 h. After treatment, the comet assay was performed to detect DNA damage. (A) Quantification of DNA damage expressed as percentage of DNA damage in the Tail and as Tail Moment (Tail length × percentage of DNA in the Tail). (B) Distribution of cells in the different intervals of values of Tail Moments. Data are averaged from two independent experiments.
